# Systematic Review of the Reliability and Reproducibility of Magnetisation Transfer Imaging in Human Cervical Spinal Cord

**DOI:** 10.3390/jcm15155768

**Published:** 2026-07-23

**Authors:** Hussein Alshaari, Mohammad Sayed, Wael Faqihi, Mohammed Asiri, Sadiyyah Almuwallad, Amani Thanyah, Nawal Al Musari, Najla Hussin, Sarah Habtar

**Affiliations:** 1 Radiological Sciences Department, College of Applied Medical Sciences, Najran University, Najran 61441, Saudi Arabia; mfsayed@nu.edu.sa (M.S.); wafaqihi@nu.edu.sa (W.F.); 441409095@nu.edu.sa (S.A.); 442301934@nu.edu.sa (A.T.); 443405249@nu.edu.sa (N.A.M.); 441301933@nu.edu.sa (N.H.); 443405464@nu.edu.sa (S.H.); 2 Health Research Center, Najran University, Najran 61441, Saudi Arabia; 3 Department of Radiological Sciences, College of Applied Medical Sciences, Taif University, Taif P.O. Box 21944, Saudi Arabia; m.asire@tu.edu.sa

**Keywords:** magnetization transfer imaging (MTI), magnetization transfer ratio (MTR), cervical spinal cord (CSC), reliability, reproducibility, repeatability

## Abstract

**Objectives:** The objective of magnetisation transfer imaging (MTI) is to quantitatively assess myelin integrity in the cervical spinal cord (CSC) using MRI technology. The magnetisation transfer ratio (MTR) has been extensively studied as a prospective biomarker for identifying microstructural alterations in both healthy and diseased tissue. This systematic review aims to assess the repeatability and reproducibility of MTI-derived MTR studies in human CSC. **Methods:** A systematic search was performed in PubMed, Scopus, and Web of Science to identify studies evaluating the reliability of MTI, specifically MTR. Studies that met the criteria presented statistical metrics including intraclass correlation coefficient (ICC), coefficient of variation (CV%), or Bland–Altman (BA) analysis. Only peer-reviewed articles in English were included. The review adhered to PRISMA 2020 guidelines, and methodological quality was evaluated using the Quality Assessment with Diverse Studies (QuADS) tool. **Results:** Thirty studies were identified; following screening, six studies satisfied the inclusion requirements. These investigations assessed the reliability and reproducibility of MTR in both healthy individuals and patients with cervical myelopathy throughout spinal levels C1–C7. The ICC values exhibited considerable variability, spanning from poor agreement to about 0.90%, depending on the region of interest. The CV% values varied from 1.3% to 6.1% in non-healthy populations and from 0.5% to 12.7% in healthy groups. The BA analysis indicated no systematic bias, characterised by relatively minimal mean differences. A quantitative meta-analysis was not conducted due to methodological differences in each study. QuADS ratings demonstrated moderate to high methodological quality (77–96%). **Conclusions:** MTR exhibits generally low ICC values over different ROIs in most of the included studies and high to medium reproducibility for the evaluation of CSC. Nonetheless, diversity in acquisition procedures, statistical reporting and ROI selection continues to be a significant limitation of our study for not reporting the meta-analysis.

## 1. Introduction

Magnetisation transfer imaging (MTI) is a quantitative magnetic resonance imaging technique that provides sensitivity to myelin integrity and, to a lesser extent, alterations in axonal microstructure [1]. By characterising the exchange between bound and free water protons within biological tissues, MTI yields a quantitative parameter known as the magnetisation transfer ratio (MTR), typically expressed as a percentage per unit (pu) [2]. MTR has been widely reported to correlate with tissue myelin content. The MTR can be expressed by the following equation:

MTR = MT_off_ − MT_on_/MT_off_

where MT_off_ reveals the single intensity of the image voxel in the absence of the MT pulse, and MT_on_ reveals the single intensity of the image voxel in the presence of the MT pulse [3]. Within the existing literature, the terms “reliability” and “reproducibility” are frequently used inconsistently, despite representing distinct measurement properties. Reliability describes the variability of repeated measurements obtained from the same subject under identical conditions, including the use of the same acquisition protocol, instrumentation, and observer, over a short time interval during which the true value of the measured parameter is assumed to remain stable. In contrast, reproducibility refers to the variability of measurements acquired from the same subject under differing conditions, such as changes in acquisition protocols, hardware, observers, or extended time intervals, during which the underlying parameter may vary. Given the technical challenges associated with quantitative imaging of the cervical spinal cord, establishing the reliability and reproducibility of MTR measurements in this region is essential. Accordingly, this systematic review aims to evaluate the reproducibility as well as reliability of MTR measurements in studies that assessed the cervical spinal cord (CSC) [3,4].

## 2. Materials and Methods

This systematic review protocol was registered on the International Prospective Register of Systematic Reviews (PROSPERO). The study protocol can be accessed at URL (25 February 2026) (https://www.crd.york.ac.uk/PROSPERO/view/CRD420261296261).

### 2.1. Eligibility Criteria

This systematic review was conducted from January to March 2026 and was reported based on the Preferred Reporting Items for Systematic Reviews and Meta-Analyses (PRISMA) 2020 statement (see Supplementary S1) [5]. Eligible studies were chosen according to the inclusion criteria described in the following sections.

### 2.2. Inclusion Criteria

Studies were eligible for inclusion if they investigated magnetisation transfer imaging (MTI), particularly the MTR biomarker, in the human CSC and reported quantitative statistical analysis of reliability and/or reproducibility. Eligible studies were required to include full-text peer-reviewed publications written in English and to report at least one statistical measure of reliability and reproducibility, such as the intraclass correlation coefficient (ICC), coefficient of variation (CV%), or Bland–Altman analysis (as shown in Table 1).

### 2.3. Exclusion Criteria

Editorials, opinion articles, conference abstracts, single-case reports, animal studies, and studies focusing exclusively on non-MTI were excluded.

### 2.4. Study Population

Studies including healthy and clinical populations that underwent MTI of the CSC were eligible for inclusion; studies conducted in animal models were excluded.

### 2.5. Source of Information

The Database of International Prospective Register of Systematic Reviews (PROSPERO) was reviewed in search of any prior systematic studies regarding the reliability and/or reproducibility of MTR in the CSC. No related systematic review was identified. An electronic search was carried out using PubMed, Scopus, and Web of Science (see Supplementary S2). The bibliographic references for each article, including full-text papers and studies that cited the referenced papers, were reviewed for highly relevant research for data extraction.

### 2.6. Research Strategy

A comprehensive literature search was undertaken throughout PubMed, Scopus, and Web of Science. Using well-known methodological search filters proposed by [6], and Ingui [7], the search strategy was created, integrating later improvements suggested by Geersing et al. (each linked using “OR”). Magnetisation transfer imaging, magnetisation transfer ratio (MTR), spinal cord, cervical spinal cord, repeatability, reliability, and reproducibility were among the search terms that were used as keywords.

### 2.7. Study Records

Studies that contained at least one of the predefined keywords in the title or abstract and fulfilled the requirements for inclusion criteria were assessed during the initial selection stage. Titles and abstracts were assessed to determine potential eligibility. Any disagreements arising during this stage were resolved through consensus. Articles considered potentially eligible were retrieved in full text and further evaluated to confirm their suitability for inclusion and synthesis. Full-text assessments were conducted independently by four reviewers, and disagreements were resolved by consensus. When agreement could not be achieved, a fifth reviewer (corresponding author) was available for clarification, although this was not required. Reasons for exclusion at the full-text screening stage were reported in accordance with the protocol of this review.

### 2.8. Data Extraction

We collected data concerning the characteristics of all included studies that reported the reliability and reproducibility assessment, and the research populations, including sample size and the health status of participants in relation to their CSC or unhealthy (with pathology) into two tables (Table 1 and Table 2). The initial table involved author names, publication year, statistical analyses employed, CSC levels assessed, region of interest (ROI), as well as outcomes of reliability and reproducibility investigations. The second table covered the technical specifications of MTI studies, specifically examining the MTR metric concerning the CSC, encompassing both healthy and pathological conditions. It detailed imaging acquisition parameters, the software utilised for the quantitative extraction of the MTR metric, and any particular pre-processing procedures implemented on the MTI data before the extraction of the MTR metric. Furthermore, the mean values of MTR measures of different ROIs were gathered, along with gender, mean age, and the selected ROI, as detailed in Table 3.

### 2.9. Outcomes and Prioritizations

#### 2.9.1. Primary Outcome

The primary outcome of this current study was to measure the statistics and metrics used in reporting reliability and/or reproducibility, such as CV, ICC, and BA analysis, in evaluating the CSC with respect to any MTR MTI-based subject-, observer-, scanner vendor-, or site-related causes, and any external validation implemented to assess reliability and/or reproducibility.

Multiple statistical metrics were employed to evaluate measurement reliability and reproducibility across the included studies, primarily including the CV%, the ICC, and the BA agreement tests. Studies that reported these reliability metrics and satisfied the predefined eligibility criteria were only considered. The CV% was used to quantify relative measurement variability and was calculated as a percentage by taking the ratio of the within-subject standard deviation to the mean value expressed. CV% values below 10% were considered indicative of low variability and acceptable measurement stability [8], whereas values between 11% and 20% reflected moderate variability, and values exceeding 20% indicated high measurement variability [9]. The ICC was widely utilised to assess test–retest reliability due to its ability to estimate the proportion of true variance relative to total observed variance, including measurement error [10]. Higher ICC values indicate improved reliability and consistency between repeated measurements. Based on commonly accepted interpretation thresholds, ICC values < 0.5 indicate poor reliability, values between 0.5 and 0.75 represent moderate reliability, values between 0.75 and 0.90 indicate good reliability, and values > 0.90 demonstrate excellent reproducibility [11]. In addition, the BA analysis was applied to assess agreement between repeated measurements by plotting the mean of paired measurements against their differences, allowing identification of systematic bias and limits of agreement [8].

#### 2.9.2. Secondary Outcomes

The contribution of multiple factors such as the impact of pre-processing steps applied prior to MTR metric extraction, MT image acquisition parameters, and MTI extraction software on the reliability or reproducibility of MTR was the secondary outcome to be assessed.

### 2.10. Evaluate the Quality of Studies

The methodological and reporting quality of the research included was evaluated using the Quality Assessment with Diverse Studies (QuADS) tool [12], which was chosen because of the methodological diversity of the studies included in this evaluation. QuADS is specifically created to assess qualitative, mixed-methods, and multimethod research in health care. It had 13 items (Table 4 and allocated a score ranging from 0 to 3 for each item (see Supplementary S3)), where 0 represents the lowest score, and 3 denotes the greatest. The upper limit for the score was established at 39. The final score was documented as a percentage [final score = (total score of each research/total score of criteria) × 100%] as reported in [3]. Each study was evaluated by the four reviewers across 13 criteria addressing theoretical framework, clarity of research aims, appropriateness of study design, sampling strategy, data collection methods, analytical justification, and discussion of strengths and limitations, and that any disagreements between four reviewers among the scoring levels were solved through discussion and consensus among the four reviewers, with a fifth reviewer (a corresponding author) available for decision in case of consensus could not be reached.

**Table 1:** Illustrates the characteristics of reliability and reproducibility of all included studies.

Study (Year)	Sample Size	Statistical Test	C-Spine Region and Levels	Reliability	Reproducibility
Lévy, Simon et al. [13] (2018)	33 Healthy volunteers	ICC	C2–C5	Single-measure ICC across WM regions (WM: 0.28 [−0.99–0.37], DC: 0.29 [−1.02–0.07], LC (LF): 0.38 [−0.94–0.53], VC: 0.35 [−1.65–0.93]). ICC across vertebral levels = (C2: 0.43); (C3: −0.30); (C4: 0.16), and (C5: 0.05).	_
Combès, Benoit et al. [14] (2019)	44 Healthy volunteers	ICC	C1–C7; C4–C6	ICC (95% CI): 0.63 (0.29–0.95); CV: 2.0–2.3%	ICC (95% CI): 0.05 (0.00–0.34); CV: 0.5–0.7%
Al-shaari et al., (2024) [15]	20 Healthy volunteers	ICC, CV%, BA	C2–C5	Single-measure ICC (95% CI): WM: 0.03 [−0.41–0.46]; DC: 0.10 [−0.35–0.51], VC: 0.39 [−0.40–0.46], LC: 0.001 [−0.43–0.61]. Average-measure ICC (95% CI): WM: 0.06 [−1.38–0.63], DC: 0.18 [−1.08–0.68], VC: 0.75 [−1.34–0.83], LC: 0.002 [−1.52–0.61]. CV%: (WM: 3%), (DC: 4%), (VC: 9%), and (LC: 4%). BA analysis: WM: Mean difference = 0.53 (−1.08–2.13); LOA = (−6.20 to 7.25), DC: Mean difference = 0.31 (−1.36–1.98); LOA = (−6.69 to 7.31), VC: Mean difference = 1.97 (−0.19–3.95); LOA = (−6.34 to 10.27), LC: Mean difference = 0.46 (−1.26–2.18); LOA = (−6.75 to 7.66).	**_**
Martin, Allan R. et al. [16] (2017)	40 healthy subjects and 18 with DCM	CV%	Rostral (C1–C3) Middle (C4–C5) Caudal (C6–C7)	CV% for Rostral (C1–C3) healthy/DCM = 2.7 ± 1.9%/1.3 ± 0.5%, for Middle (C4–C5) healthy/DCM = 3.2 ± 3.0%/6.1 ± 0.9%, for Caudal (C6–C7) healthy/DCM = 4.4 ± 3.8%, 3.1 ± 3.9%	**_**
Yiannakas, Marios C. et al. [17] (2012)	10 healthy volunteers	ICC, CV%	C2–C3	TCA ICC = 0.5 TCA CV% = 0.7% TGMA ICC = 5.4 TGMA CV% = 6.5%	TCA ICC = 0.9 TCA CV% = 0.5% TGMA ICC = 0.7 TGMA CV% = 12.7%
Boudreau, Mathieu et al., (2025) [18]	6 healthy volunteers	ICC, CV%	C2–C5	CV% = 5.4%	ICC: 0.2, CV = 4.6%

WM = White Matter; DC = Dorsal Column; VC = Ventral Column; LC = Lateral Column; DCM = Degenerative Cervical Myelopathy; TCA = Total Cord cross-sectional Area; TGMA = Total Grey Matter area; ICC = Intraclass Correlation Coefficient; CV = Coefficient of Variation; BA = Bland–Altman; LOA = Limits of Agreement.

**Table 2:** Shows acquisition details of all included studies.

Study (Year)	Filed Strength (T)	Scanner Make	Acquisition	Slice Thickness (mm)	TR (ms)	TE (ms)	NO. Slices	Signal Averages	Scan Time	Voxel Size (mm)	FOV (mm)	Software for MTR Metrics Extraction; Version
Lévy et al., (2018) [13]	3	Siemens	Axial	-	3000	19	17	_	18 min	0.9 × 0.9	192 × 192	SCT; 2.2.3
Combès et al., (2019) [14]	3	Siemens	Axial	3	38	3.57	-	_	14 min	0.7 × 0.7	_	SCT; 3.0, Anima; 2.3
H. Al-shaari et al., (2024) [15]	3	Siemens	Axial	5	35	313.	22	1	2:39 s	0.9 × 0.9	86 × 86	SCT; v5.4
Martin et al., (2017) [16]	3	General Electric (GE)	Axial	5	32	5.9	-	-	3:45 s	1 × 1	190 × 190	SCT; 2.3
Yiannakas et al., (2012) [17]	3	Philips Achieva	Axial	5	36	TE1/TE2 = 3.5/5.9	22	2	23 min	0.5 × 0.5	240 × 180	JIM; 6.0 (Xinapse; (http://www.xinapse.com), FSL (http://www.fmrib.ox.ac.uk/fsl/)
Boudreau, Mathieu et al., (2025) [18]	3	Siemens	Axial	5	15	3.13	-	2	18:22 s	0.9 × 0.9	180 × 180	SCT; 5.6

**Table 3:** Illustrates mean and standard deviation (SD) values of MTR measured as percentage unit (pu) in healthy and non-healthy cervical spinal cord based on c-spine level and selected ROI of each study.

Author (Population)	Gender (Mean Age)	C-Spine Level	MTR Metric Mean ± SD and/or (95% CI)
Lévy, Simon et al. [13] (2018) (33 healthy adults)	Not reported	C2–C5	C2: 46.83 ± 1.52 C3: 45.78 ± 1.38 C4: 44.87 ± 1.55 C5: 44.02 ± 1.90
Combès, Benoit et al. [14] (2019) (44 healthy volunteers)	20 males (32.8 ± 7.6)	C1–C7	34.6 ± 0.9
Al-shaari et al., [15] (2024) (20 healthy volunteers)	10 male (33.9 ± 3.6); 10 female (47.6 ± 14.4)	C2–C5	WM: 49.20 (95% CI 48.37–50.02); DC: 48.64 (47.72–49.56); VC: 49.47 (48.43–50.0); LC: 49.14 (48.27–49.10)
Martin, Allan R. et al. [16] (2017) (40 Healthy; 18 DCM)	In healthy: 21 male; 19 female (47.1 ± 15.3). In DCM: 11 male; 7 female (56.4 ± 11)	C1–C7	39.5 ± 2.1 (healthy); 37.9 ± 2.6 (DCM)
Yiannakas, Marios C. et al. [17] (2012) (10 healthy volunteers)	10 male (33; range 22 to 51.7)	C2–C3	WM-MTR = 51.4 ± 1.5; GM-MTR = 49.7 ± 1.6
Boudreau, Mathieu et al. [18] (6 healthy participants)	3 male; 3 female (range 31 to 47)	C2–C5	Not reported

WM = White Matter; GM = Grey Matter; DC = Dorsal Column; VC = Ventral Column; LC = Lateral Column; DCM = Degenerative Cervical Myelopathy; MTR = Magnetisation Transfer Imaging; SD = Standard Deviation; 95% CI = Confidence Interval.

**Table 4:** Shows the Quality assessment with diverse studies (QuADS) items and scores for each included study.

Articles	Al-Shaari et al., [15]	Martin, Allan R. et al. [16]	Combès, Benoit et al. [14]	Boudreau, Mathieu et al. [18]	Lévy, Simon et al. [13]	Yiannakas, Marios C. et al. [17]
1. Theoretical or conceptual underpinning to the research	3	3	3	3	3	3
2. Statement of research aim/s	3	3	3	3	3	3
3. Clear description of research setting and target population	3	3	3	3	3	3
4. The study design is appropriate to address the stated research aim/s	3	3	3	3	3	3
5. Appropriate sampling to address the research aim/s	2	2	2	3	2	2
6. Rationale for choice of data collection tool/s	2	3	3	3	3	3
7. The format and content of the data collection tool is appropriate to address the stated research aim/s	3	3	3	3	3	3
8. Description of data collection procedure	3	3	3	3	3	3
9. Recruitment data provided	2	2	2	2	3	2
10. Justification for analytic method selected	3	3	3	3	3	3
11. The method of analysis was appropriate to answer the research aim/s	3	3	3	3	3	3
12. Evidence that the research stakeholders have been considered in research design or conduct	0	0	0	2	3	2
13. Strengths and limitations critically discussed	3	3	2	3	3	3
Total scores	30	34	30	36	34	33
Score as Percentage	77%	85%	77%	96%	87.2%	84.6%

## 3. Results

### 3.1. Literature Search and Study Selection

**Figure 1:**
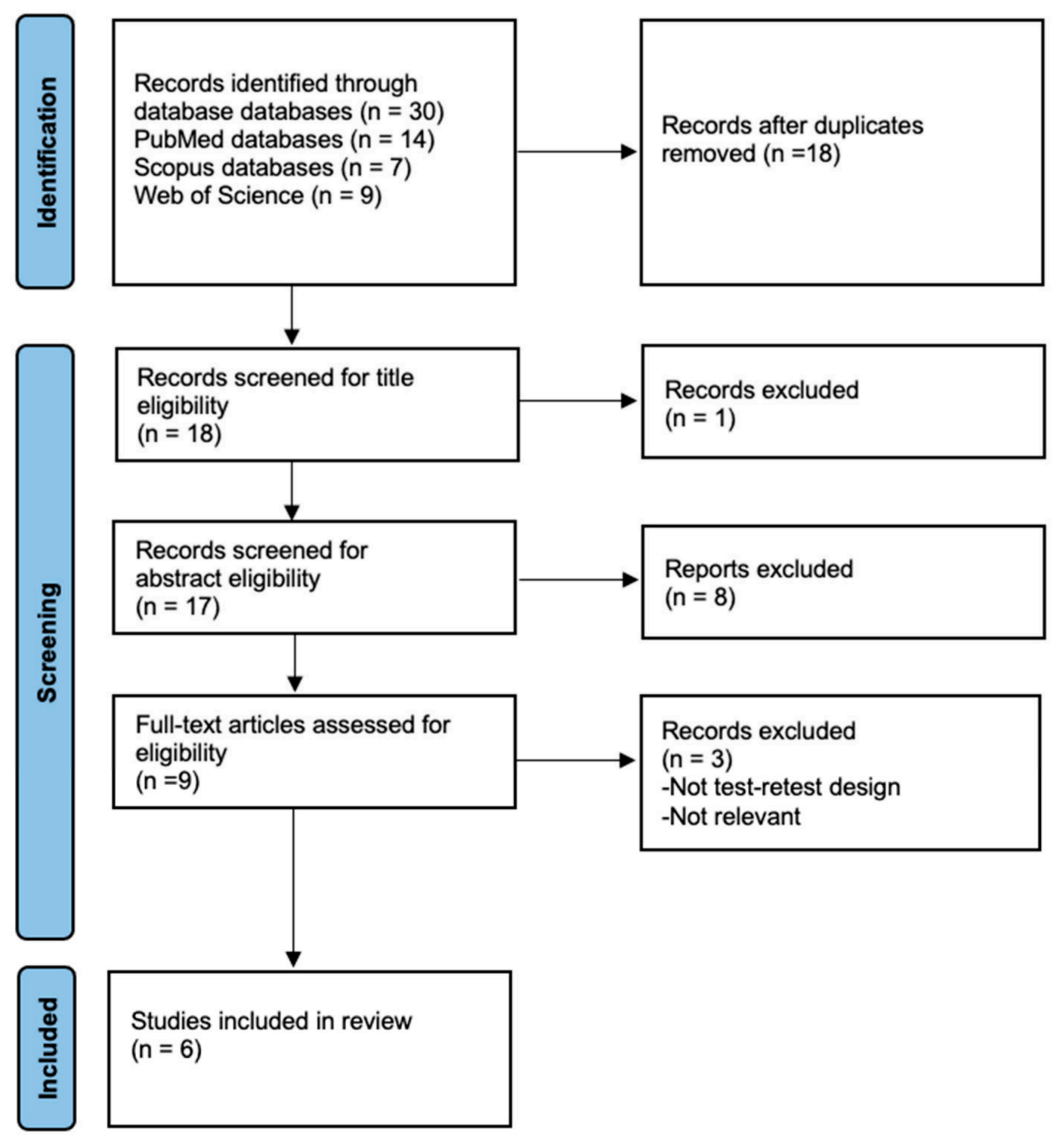
Shows the flow chart graph of the study search strategy and the process of selection using the (PRISMA) guideline. PRISMA stands for the Preferred Reporting Items for Systematic Reviews and Meta-Analyses.

Figure 1 illustrates the flow diagram summary as suggested by the PRISMA checklist [5]. The database search across PubMed, Scopus, and Web of Science identified 30 records. After removal of duplicates and initial screening, nine full-text articles were assessed for eligibility. Three studies were excluded at the full-text stage because they investigated other topics rather than magnetisation transfer imaging (MTI) or the MTR, which were the focus of the present review. Consequently, six studies met the predefined inclusion criteria and were included in the final qualitative synthesis.

### 3.2. Characteristics of the Included Studies

The methodological characteristics and imaging parameters of data acquisition of the included studies are summarised in Table 1 and Table 2. The six studies examined the reliability and reproducibility of MTR measurements in the CSC in both healthy and non-healthy participants. Sample sizes across the included studies ranged from 6 to 44 for healthy participants, with one study additionally including 18 DCM patients. The investigated spinal cord regions varied between studies, covering cervical levels from C1 to C7, although most studies focused on upper to mid-cervical segments (commonly C2–C5). All studies were conducted using a 3-Tesla MRI system, although acquisition parameters differed across studies, including variations in repetition time (TR), echo time (TE), voxel size, field of view (FOV), and slice thickness. Regions of interest (ROIs) were analysed across studies included whole spinal cord measurements as well as tissue-specific regions such as white matter (WM), grey matter (GM), and specific white-matter subregions such as dorsal column (DC), ventral column (VC), and lateral column (LC), and An additional methodological factor influencing reliability assessments was the time interval between repeated scans, which varied across studies from short within-session intervals (approximately one hour) to longer test–retest intervals of up to three months.

### 3.3. Reliability and Reproducibility of MTR Measurements

The reliability and reproducibility of MTR measurements were assessed using several statistical metrics, primarily including ICC, CV%, and BA agreement evaluation. Across the included studies, ICC values demonstrated substantial variability, ranging from poor agreement to values approaching 0.90, depending on the spinal cord region, measurement protocol, and analysis method. In several studies, single-measure ICC values were relatively low, whereas average-measure ICC values indicated improved reliability, suggesting that averaging repeated measurements may enhance measurement stability. The CV% values reported across studies were generally low, indicating relatively stable MTR measurements. In healthy cohorts, CV% values ranged approximately from 0.7% to 9%, whereas studies including patients with DCM reported CV values ranging from 1.3% to 6.1%. BA analyses, which provide direct insight into systematic bias and limits of agreement between measurements, performed in one study demonstrated small mean differences between repeated measurements, with no consistent evidence of systematic bias. However, variability in limits of agreement was observed across different CSC regions.

Due to methodological heterogeneity across studies, including differences in imaging acquisition parameters, ROI segmentation strategies, and statistical analysis approaches, a quantitative meta-analysis was not evaluated. Overall, the available evidence indicates that MTR measurements in the CSC demonstrate generally low ICC values over different ROIs in most of the included studies and high to medium reproducibility values for the evaluation of CSC, although measurement variability remains influenced by acquisition protocols, spinal cord levels examined, and region-of-interest definition.

### 3.4. Normative Values of MTR

Normative MTR values in included studies (Table 3) ranged from 34.6 ± 0.9 pu in whole-cord measurements [14] to 51.4 ± 1.5 pu in WM at C2–C3 [17]. WM consistently showed higher MTR values than GM, reflecting its greater myelin content [19]. In a single study with a DCM cohort, patients had somewhat lower MTR values (37.9 ± 2.6 pu) than healthy controls (39.5 ± 2.1 pu), indicating that MTR is vulnerable to pathological microstructural alterations caused by cord compression [16].

### 3.5. Quality Assessment

The methodological quality of the included studies was found to be varied according to the QuADS criteria (Table 4). The quality of the investigations was mixed, with moderate to high percentages. Most papers achieved relatively moderate to high methodological quality scores, ranging from approximately 77% to 96%. These studies exhibited well-justified analytic methods, appropriate study designs, clearly stated objectives, and robust conceptual frameworks. Nevertheless, minor deficiencies were identified in numerous domains. Limitations in recruitment detail and sampling justification were identified in certain studies, particularly in pilot or technical feasibility designs. Variability was also noted in the reporting of the contribution of stakeholders to the design and research process. Many of the evaluated studies did not provide any explicit evidence of stakeholder engagement, which led to lower scores for this particular QuADS item. The overall quality of the methodology of the included studies was considered high, despite these limitations. The appropriateness of the study design, data collection procedures, analytic justification, and critical discussion of strengths and limitations were consistently observed as strengths.

## 4. Discussion

In general, the small number of available studies limits the strength and generalisability of the conclusions of the current review. The overall reliability and reproducibility metrics of MTR differed among the included studies and showed regional and metric-dependent variability, with CV% and BA tests representing acceptable relative variability, but ICC values showed considerable inconsistency, demonstrating poor reliability in some CSC levels and ROIs.

The use of MTI for assessing the CSC level is challenging due to several technical challenges that remain in quantitative spinal cord MRI, such as susceptibility artefacts, artefacts due to physiological motion from cerebrospinal fluid (CSF) pulsation and respiration, and variability in acquisition parameters [15]. Moreover, the cord-restricted cross-sectional area measures only 1 cm in diameter. Therefore, submillimeter spatial resolution is necessary for distinguishing between GM and WM structures. Moreover, several factors might limit image quality, including cardiac pulsations and partial volume effects (PVE) between WM and surrounding CSF [3,15,20,21]. Further, diversity in acquisition protocol, statistical reporting, and ROI selection of all included studies should also be considered as important key limitations that might contribute to the overall reporting of reliability and reproducibility. All these factors may negatively result in measurement variability of MTR. Additionally, differences in scanner hardware, image resolution, segmentation methods, and ROI may also explain discrepancies in reliability and reproducibility metrics reported across studies. Moreover, a number of strategies have been employed in a number of studies to address the MTI challenge in CSC imaging, such as cardiac gating to mitigate the impact of heart motion and CSF pulsation during the scan. Additionally, a reduced voxel size has been used to reduce PVE during data post-processing.

This systematic review synthesised evidence from six studies investigating the reliability and reproducibility of MTR measurements in both healthy individuals and patients with CSC pathology. In diseases such as DCM, MTI has been highlighted as one of the developing quantitative imaging techniques for objective profiling along with other novel diagnostic and prognostic tools [22]. MTR measures in DCM are becoming more clinically relevant as evidence of their predictive efficacy develops. Higher preoperative MTR values have been linked to better postoperative improvements in modified Japanese Orthopaedic Association (mJOA) ratings and health-related quality-of-life outcomes following surgical intervention [23]. Furthermore, tract-specific reductions in MTR were revealed to be correlated with identified patterns of neurological impairment and the severity of disease in DCM [23]. These findings demonstrate the ability of MTR to provide a supplementary, objective categorisation of DCM patients beyond what the mJOA score simply can convey, especially since inter-rater reliability of the mJOA has been demonstrated to be imperfect near severity-category thresholds [24]. The AO Spine Knowledge Forum on Spinal Cord Injury has emphasised the need for a multimodal imaging strategy for DCM, recognising that no single modality can properly characterise spinal cord microstructural alterations [25,26]. Within this context, MTI may contribute additional information to conventional MRI and other quantitative techniques such as DTI. However, because the studies included in this review did not directly compare MTI to other imaging modalities, no inferences can be reached about its relative performance. Future comparison studies comparing the reproducibility and clinical value of MTI to other imaging modalities are necessary.

The findings of the current review suggest that MTR demonstrated commonly low ICC values over the examined ROIs in most of the included studies and high to medium reproducibility for the assessment of the CSC, although variability remains influenced by factors such as imaging protocols, anatomical regions examined, statistical methods applied and methodological differences across the included studies. Across the included studies, several statistical metrics were used to evaluate measurement reliability, including the ICC, CV%, and Bland–Altman analysis. Considerable variability in ICC values was reported across studies and regions of interest. For instance, Lévy et al. [13] reported low to moderate ICC values across WM regions in the CSC between C2 and C5. Similarly, Al-shaari et al. [15] demonstrated generally low single-measure ICC values across WM and WM subregions, although improved reliability was observed for average measurements in certain regions such as the VC. These findings indicate that while individual measurements may exhibit limited reliability, averaging measurements may improve the reliability in quantitative spinal cord imaging. A closer comparison of the studies presented in Table 1 highlights an important difference in measurement reliability across studies. In terms of reproducibility assessment (CV%), Martin et al. (2017) [16] demonstrated relatively high reproducibility metrics compared with the other studies, which ranged approximately between 2.7% and 4.4% in healthy participants and between 1.3% and 6.1% in patients with DCM, indicating good measurement precision. Similarly, Combès et al. [14] reported low overall variability in MTR measurements across multiple scanners in a multicenter study, with an overall CV of approximately 3%. The overall CV% of these studies suggest stable measurement precision, and the differences in reproducibility among the included studies may partly be explained by methodological variations reported in Table 2, including voxel size, slice thickness, acquisition time, and image processing pipelines. For the consistency evaluation using BA, only one study [15] assessed the agreement between repeated MTR measurements. Unlike the ICC, which evaluates reliability, and CV, which reflects relative variability, BA analysis provides direct insight into systematic bias and limits of agreement between measurements. The study demonstrated small mean differences, indicating minimal systematic bias, along with relatively narrow limits of agreement despite variability in the ICC values, suggesting that measurement differences remain clinically acceptable. Therefore, BA analysis complements the ICC and the CV by strengthening the overall interpretation of measurement reliability and agreement [15] and is suggested to be involved in future reliability assessment of MTI studies.

The results of the current review also indicate that when standardised acquisition protocols are applied, the MTR measurements can demonstrate acceptable reproducibility across different imaging sites and scanner environments. In addition, other factors such as tissue-specific analysis of spinal cord structures appear to play an important role in improving measurement precision. Yiannakas et al. [17] demonstrated the probability of segmenting GM and WM within the superior levels of CSC. They showed that WM-MTR values were consistently higher than GM values, reflecting differences in myelin content. Such tissue-specific quantification may yield more sensitive biomarkers for detecting microstructural alterations in spinal cord disorders. Furthermore, despite the VC exhibiting the highest average ICC in Al-shaari et al.’s study [15], it also produced the greatest CV and mean differences among the ROIs. This contrasts with the study by Lévy et al. [13], which indicated that the low dependability in C3 (ICC = −0.3) resulted from significant variation among repeated assessments. Various mechanisms may account for the low single and average ICCs for MTR reported in prior MT studies, including variations in coil characteristics, magnetic field strength, sequence parameters, and fluctuations in the power of saturation pulses and offset frequency, as reported in Al-shaari et al.’s study [15]. Moreover, the significant disparity noted for a limited number of participants, or sample size, in all included studies may have substantially influenced the ICC values. The elevated values noted in the VC regions and the substantial disparity between individual and mean values in Al-shaari et al. [15] may resulted from that the VC being derived from exceedingly small ROI tract volumes delineated by the “Polytechnique”, “Aix-Marseille University” and “Montreal Neurological Institute” (PAM50) atlas [27]; consequently, this region may be more vulnerable to PVE than the WM subregions.

In comparison to other imaging modalities, the reliability and reproducibility of MTR in the CSC appears to be equivalent to that of other quantitative MRI biomarkers. A systematic review of reliability and reproducibility of diffusion tensor imaging (DTI) found that ICC values ranged from poor to excellent depending on the metric and ROI, while CV values and BA analyses generally indicated good reproducibility and agreement, respectively; however, significant methodological heterogeneity precluded quantitative meta-analysis [3]. Similarly, for myelin water imaging (MWI), Ljungberg et al. (2017) [28] found a mean CV of 6.1% in healthy CSC participants, indicating acceptable scan-rescan reliability. Together, these findings show that ROI-dependent variability and inconsistent ICC estimations, despite generally positive CV and BA outcomes, are typical aspects of quantitative spinal cord MRI approaches rather than constraints unique to MTR.

A meta-analysis was not performed due to methodological heterogeneity sources. First, there was significant variation in acquisition protocols among studies. Repetition time (TR), echo time (TE), voxel size, slice thickness, and scan duration were among the parameters that varied significantly among the included investigations. For example, in-plane voxel sizes ranged from 0.5 × 0.5 mm to 1 × 1 mm. These variations are not insignificant: MTR measurements in the CSC are especially sensitive to acquisition parameters because the cord’s small cross-sectional diameter (about 8–12 mm at cervical levels) means that even slight variations in voxel size can significantly change the degree of PVE with surrounding tissue boundaries and CSF. PVE is a well-documented source of measurement variability in spinal cord quantitative MRI, especially when imaging smaller WM subregions like the DCs and LCs, where voxels at the cord periphery may contain signal contributions from both tissue and CSF, artificially reducing or inflating MTR values and degrading both accuracy and reproducibility [29]. Moreover, differences in MT pulse design (e.g., frequency offset, flip angle, pulse shape) between scanner vendors (Siemens, GE, Philips) mean that absolute MTR values are not directly comparable across studies, even when the same nominal protocol is used, because MTR is a semi-quantitative metric that lacks the physical standardisation of quantitative magnetisation transfer (qMT) parameters. This is a well-known limitation of the field, which inspired the creation of the spine-generic protocol [30]. Second, the statistical analysis methodologies were not consistent among investigations. The included studies used a mix of single-measure ICC, average-measure ICC, CV%, and BA analyses, with inconsistent reporting of confidence intervals, ICC model type (one-way or two-way, random or mixed effects), and scoring type definition (agreement versus consistency). This variation in statistical reporting is a well-known obstacle to synthesis in reliability systematic reviews, as different ICC model configurations might provide significantly different numerical estimates from the same dataset. None of the included research consistently followed a uniform reporting structure, such as the GRAPPA (Guidelines for Reporting Reliability and Agreement research) recommendations, restricting cross-study comparability [31]. Third, population variability between included studies—ranging from studies with only healthy volunteers to those with patients with DCM—creates an additional limitation to pooling. Pathological alterations in the cervical spinal cord in DCM, such as cord compression, oedema, and myelomalacia, alter MTR values and their spatial distribution, potentially altering both the absolute measurement and its variability on subsequent scans. Studies conducted in mixed healthy/DCM cohorts are thus not directly comparable to studies conducted in healthy volunteers alone in terms of expected measurement reliability. Fourth, ROI segmentation methods differed greatly between studies, from whole-cord analysis to tissue-specific segmentation of WM, GM, and individual WM columns utilising various software platforms. This methodological heterogeneity most likely led to the variation in reported ICC values. Because ICC is influenced by between-subject variability, lower ICCs may occur in homogeneous cohorts or small ROIs with a narrow range of real values, even if measurement error is acceptable [4,32]. As a result, lower ICC values should not be interpreted as poor measurement reliability, but rather in terms of sample characteristics and ROI size. This is especially important when comparing research with diverse participant demographics and segmentation methodologies [32].

## 5. Conclusions

This systematic review evaluated the reliability and reproducibility of MTR measurements derived from MTI in the human CSC. The small number of included studies has limited the strength and generalizability of the conclusion and should be interpreted as provisional rather than definitive. Overall, the reliability and reproducibility metrics of MTR varied across studies and are metric- and region-dependent, with CV% and BA analyses representing acceptable relative variability; however, ICC values show considerable inconsistency, illustrating poor reliability in some cervical levels and CSC ROIs. This discrepancy could depend on different factors such as imaging protocols, CSC levels examined, statistical reliability and reproducibility tests, and ROI segmentation strategies. Studies reporting lower CV, moderate ICC and minimal BA differences suggest that standardised acquisition protocols, multicenter evaluation and consistent ROI definitions may improve measurement consistency and the overall reporting of reliability and reproducibility.

### Limitations and Future Directions

Several limitations should be considered when interpreting the findings of this systematic review. First, the number of eligible studies was relatively small: only six met the inclusion criteria, which may limit the generalizability of the findings. Second, substantial methodological heterogeneity was observed across the included studies, including differences in MRI acquisition parameters, CSC levels examined, ROI segmentation strategies, and statistical approaches used to evaluate repeatability and reproducibility. These methodological differences limited direct comparison between studies and prevented the performance of a quantitative meta-analysis. Future studies might be explored with a larger number of studies and a larger number of participants. In addition, adjusting a specific protocol for CSC may improve the reliability and reproducibility of future studies.

## Data Availability

The data supporting the conclusions of this article will be made available by the authors on request.

## Supplementary Materials

The following supporting information can be downloaded at: https://www.mdpi.com/article/10.3390/jcm15155768/s1, S1: the PRISMA checklist; S2: Study Research Keywords; S3: QuADS Criteria for quality assessment.

## References

[1] Edwards L.J., Kirilina E., Mohammadi S., Weiskopf N. (2018). Microstructural imaging of human neocortex in vivo. Neuroimage.

[2] Filippi M. (2015). Oxford Textbook of Neuroimaging.

[3] Al-Shaari H., R M., CJ H. (2023). A systematic review of repeatability and reproducibility studies of diffusion tensor imaging of cervical spinal cord. Br. J. Radiol..

[4] Bartlett J.W., Frost C. (2008). Reliability, repeatability and reproducibility: Analysis of measurement errors in continuous variables. Ultrasound Obstet. Gynecol..

[5] Page M., McKenzie J., Bossuyt P., Boutron I., Hoffmann T., Mulrow C. (2021). The PRISMA 2020 statement: An updated guideline for reporting systematic reviews [Internet]. BMJ.

[6] Haynes R.B., McKibbon K.A., Wilczynski N.L., Walter S.D., Werre S.R. (2005). Optimal search strategies for retrieving scientifically strong studies of treatment from Medline: Analytical survey. BMJ.

[7] Ingui B.J., Rogers M.A. (2001). Searching for clinical prediction rules in MEDLINE. J. Am. Med. Inform. Assoc..

[8] Marenco S., Rawlings R., Rohde G.K., Barnett A.S., Honea R.A., Pierpaoli C., Weinberger D.R. (2006). Regional distribution of measurement error in diffusion tensor imaging. Psychiatry Res. Neuroimaging.

[9] Carlson H.L., Laliberté C., Brooks B.L., Hodge J., Kirton A., Bello-Espinosa L., Hader W., Sherman E.M. (2014). Reliability and variability of diffusion tensor imaging (DTI) tractography in pediatric epilepsy. Epilepsy Behav..

[10] Shrout P.E., Fleiss J.L. (1979). Intraclass correlations: Uses in assessing rater reliability. Psychol. Bull..

[11] Cicchetti D.V. (1994). Guidelines, criteria, and rules of thumb for evaluating normed and standardized assessment instruments in psychology. Psychol. Assess..

[12] Harrison R., Jones B., Gardener P., Lawton R. (2021). Quality assessment with diverse studies (QuADS): An appraisal tool for methodological and reporting quality in systematic reviews of mixed-or multi-method studies. BMC Health Serv. Res..

[13] Lévy S., Guertin M.-C., Khatibi A., Mezer A., Martinu K., Chen J.-I., Stikov N., Rainville P., Cohen-Adad J. (2018). Test-retest reliability of myelin imaging in the human spinal cord: Measurement errors versus region-and aging-induced variations. PLoS ONE.

[14] Combès B., Monteau L., Bannier E., Callot V., Labauge P., Ayrignac X., Carra Dallière C., Pelletier J., Maarouf A., de Seze J. (2019). Measurement of magnetization transfer ratio (MTR) from cervical spinal cord: Multicenter reproducibility and variability. J. Magn. Reson. Imaging.

[15] Al-Shaari H., Heales C., Fulford J. (2024). Within-participants reliability and measurement error of magnetization transfer imaging determinations within the healthy cervical spinal cord. Radiography.

[16] Martin A., De Leener B., Cohen-Adad J., Cadotte D., Kalsi-Ryan S., Lange S., Tetreault L., Nouri A., Crawley A., Mikulis D. (2017). Clinically feasible microstructural MRI to quantify cervical spinal cord tissue injury using DTI, MT, and T2*-weighted imaging: Assessment of normative data and reliability. Am. J. Neuroradiol..

[17] Yiannakas M.C., Kearney H., Samson R., Chard D.T., Ciccarelli O., Miller D.H., Wheeler-Kingshott C.A. (2012). Feasibility of grey matter and white matter segmentation of the upper cervical cord in vivo: A pilot study with application to magnetisation transfer measurements. Neuroimage.

[18] Boudreau M., Karakuzu A., Boré A., Pinsard B., Zelenkovski K., Alonso-Ortiz E., Boyle J., Bellec L., Cohen-Adad J. (2025). Longitudinal reproducibility of brain and spinal cord quantitative MRI biomarkers. Imaging Neurosci..

[19] Schmierer K., Scaravilli F., Altmann D.R., Barker G.J., Miller D.H. (2004). Magnetization transfer ratio and myelin in postmortem multiple sclerosis brain. Ann. Neurol..

[20] Oh J., Chen M., Cybulsky K., Suthiphosuwan S., Seyman E., Dewey B., Diener-West M., van Zijl P., Prince J., Reich D.S. (2020). Five-year longitudinal changes in quantitative spinal cord MRI in multiple sclerosis. Mult. Scler. J..

[21] Smith S.A., Xavier G., Ali F., Craig K.J., Gerald V.R., Hugo W.M., Peter Cm Z. (2005). Magnetization transfer weighted imaging in the upper cervical spinal cord using cerebrospinal fluid as intersubject normalization reference (MTCSF imaging). Magn. Reson. Med. Off. J. Int. Soc. Magn. Reson. Med..

[22] Ganau M., Holly L.T., Mizuno J., Fehlings M.G. (2018). Future directions and new technologies for the management of degenerative cervical myelopathy. Neurosurg. Clin..

[23] Viswanathan V.K., Shetty A.P., Jakkepally S., Kanna R.M., Rajasekaran S. (2020). Symptomatic unilateral pediculolysis associated with contralateral spondylolysis and spondylolisthesis in adults—Case report and review of literature. World Neurosurg..

[24] Martin A.R., Jentzsch T., Wilson J.R., Moghaddamjou A., Jiang F., Rienmueller A., Badhiwala J.H., Akbar M.A., Nater A., Oitment C. (2021). Inter-rater reliability of the modified Japanese orthopedic association score in degenerative cervical myelopathy: A cross-sectional study. Spine.

[25] Zipser C.M., Fehlings M.G., Margetis K., Curt A., Betz M., Sadler I., Tetreault L., Davies B.M., Committee A.S.R.D.S., Group D.C.W. (2022). Proposing a framework to understand the role of imaging in degenerative cervical myelopathy: Enhancement of MRI protocols needed for accurate diagnosis and evaluation. Spine.

[26] Martin A.R., Tetreault L., Nouri A., Curt A., Freund P., Rahimi-Movaghar V., Wilson J.R., Fehlings M.G., Kwon B.K., Harrop J.S. (2022). Imaging and electrophysiology for degenerative cervical myelopathy [AO spine RECODE-DCM research priority number 9]. Glob. Spine J..

[27] De Leener B., Fonov V.S., Collins D.L., Callot V., Stikov N., Cohen-Adad J. (2018). PAM50: Unbiased multimodal template of the brainstem and spinal cord aligned with the ICBM152 space. Neuroimage.

[28] Ljungberg E., Vavasour I., Tam R., Yoo Y., Rauscher A., Li D.K., Traboulsee A., MacKay A., Kolind S. (2017). Rapid myelin water imaging in human cervical spinal cord. Magn. Reson. Med..

[29] Lévy S., Benhamou M., Naaman C., Rainville P., Callot V., Cohen-Adad J. (2015). White matter atlas of the human spinal cord with estimation of partial volume effect. Neuroimage.

[30] Cohen-Adad J., Alonso-Ortiz E., Abramovic M., Arneitz C., Atcheson N., Barlow L., Barry R.L., Barth M., Battiston M., Büchel C. (2021). Generic acquisition protocol for quantitative MRI of the spinal cord. Nat. Protoc..

[31] Kottner J., Audigé L., Brorson S., Donner A., Gajewski B.J., Hróbjartsson A., Roberts C., Shoukri M., Streiner D.L. (2011). Guidelines for reporting reliability and agreement studies (GRRAS) were proposed. Int. J. Nurs. Stud..

[32] Koo T.K., Li M.Y. (2016). A guideline of selecting and reporting intraclass correlation coefficients for reliability research. J. Chiropr. Med..

