# Supplementary material for: Systematic Review of the Reliability and Reproducibility of Magnetisation Transfer Imaging in Human Cervical Spinal Cord
Source: J Clin Med. 2026 Jul 23;15(15):5768. doi: 10.3390/jcm15155768 (PMC13466279; doi:10.3390/jcm15155768)
Supplement: Supplementary file 1 [file jcm-15-05768-s001.zip › jcm-4310315-supplementary.pdf]

S1

Section and Topic	Item #	Checklist item	Location where item is reported
**TITLE**			
Title	1	Identify the report as a systematic review.	1
**ABSTRACT**			
Abstract	2	See the PRISMA 2020 for Abstracts checklist.	1
**INTRODUCTION**			
Rationale	3	Describe the rationale for the review in the context of existing knowledge.	2
Objectives	4	Provide an explicit statement of the objective(s) or question(s) the review addresses.	2
**METHODS**			
Eligibility criteria	5	Specify the inclusion and exclusion criteria for the review and how studies were grouped for the syntheses.	2
Information sources	6	Specify all databases, registers, websites, organisations, reference lists and other sources searched or consulted to identify studies. Specify the date when each source was last searched or consulted.	3
Search strategy	7	Present the full search strategies for all databases, registers and websites, including any filters and limits used.	3
Selection process	8	Specify the methods used to decide whether a study met the inclusion criteria of the review, including how many reviewers screened each record and each report retrieved, whether they worked independently, and if applicable, details of automation tools used in the process.	3
Data collection process	9	Specify the methods used to collect data from reports, including how many reviewers collected data from each report, whether they worked independently, any processes for obtaining or confirming data from study investigators, and if applicable, details of automation tools used in the process.	3
Data items	10a	List and define all outcomes for which data were sought. Specify whether all results that were compatible with each outcome domain in each study were sought (e.g. for all measures, time points, analyses), and if not, the methods used to decide which results to collect.	3-4
	10b	List and define all other variables for which data were sought (e.g. participant and intervention characteristics, funding sources). Describe any	3-4

Section and Topic	Item #	Checklist item	Location where item is reported
		assumptions made about any missing or unclear information.	
Study risk of bias assessment	11	Specify the methods used to assess risk of bias in the included studies, including details of the tool(s) used, how many reviewers assessed each study and whether they worked independently, and if applicable, details of automation tools used in the process.	4
Effect measures	12	Specify for each outcome the effect measure(s) (e.g. risk ratio, mean difference) used in the synthesis or presentation of results.	N/A
Synthesis meth-ods	13a	Describe the processes used to decide which studies were eligible for each synthesis (e.g. tabulating the study intervention characteristics and comparing against the planned groups for each synthesis (item #5)).	3
	13b	Describe any methods required to prepare the data for presentation or synthesis, such as handling of missing summary statistics, or data con-versions.	N/A
	13c	Describe any methods used to tabulate or visually display results of individual studies and syntheses.	3
	13d	Describe any methods used to synthesize results and provide a rationale for the choice(s). If meta-analysis was performed, describe the model(s), method(s) to identify the presence and extent of statistical heterogeneity, and software package(s) used.	N/A
	13e	Describe any methods used to explore possible causes of heterogeneity among study results (e.g. subgroup analysis, meta-regression).	N/A
	13f	Describe any sensitivity analyses conducted to assess robustness of the synthesized results.	N/A
Reporting bias assessment	14	Describe any methods used to assess risk of bias due to missing results in a synthesis (arising from reporting biases).	4
Certainty assess-ment	15	Describe any methods used to assess certainty (or confidence) in the body of evidence for an outcome.	N/A
**RESULTS**			
Study selection	16a	Describe the results of the search and selection process, from the number of records identified in the search to the number of studies included in the review, ideally using a flow diagram.	12
	16b	Cite studies that might appear to meet the inclusion criteria, but which were excluded, and explain why they were excluded.	12

Section and Topic	Item #	Checklist item	Location where item is reported
Study characteristics	17	Cite each included study and present its characteristics.	12
Risk of bias in studies	18	Present assessments of risk of bias for each included study.	13
Results of individual studies	19	For all outcomes, present, for each study: (a) summary statistics for each group (where appropriate) and (b) an effect estimate and its precision (e.g. confidence/credible interval), ideally using structured tables or plots.	5-10
Results of syntheses	20a	For each synthesis, briefly summarise the characteristics and risk of bias among contributing studies.	12
	20b	Present results of all statistical syntheses conducted. If meta-analysis was done, present for each the summary estimate and its precision (e.g. confidence/credible interval) and measures of statistical heterogeneity. If comparing groups, describe the direction of the effect.	N/A
	20c	Present results of all investigations of possible causes of heterogeneity among study results.	N/A
	20d	Present results of all sensitivity analyses conducted to assess the robustness of the synthesized results.	N/A
Reporting biases	21	Present assessments of risk of bias due to missing results (arising from reporting biases) for each synthesis assessed.	13
Certainty of evidence	22	Present assessments of certainty (or confidence) in the body of evidence for each outcome assessed.	N/A
**DISCUSSION**			
Discussion	23a	Provide a general interpretation of the results in the context of other evidence.	13
	23b	Discuss any limitations of the evidence included in the review.	13-16
	23c	Discuss any limitations of the review processes used.	16
	23d	Discuss implications of the results for practice, policy, and future research.	16
**OTHER INFORMATION**			
Registration and	24a	Provide registration information for the review, including register name and registration number, or state that the review was not registered.	2

Section and Topic	Item #	Checklist item	Location where item is reported
protocol	24b	Indicate where the review protocol can be accessed, or state that a protocol was not prepared.	2
	24c	Describe and explain any amendments to information provided at registration or in the protocol.	N/A
Support	25	Describe sources of financial or non-financial support for the review, and the role of the funders or sponsors in the review.	16
Competing interests	26	Declare any competing interests of review authors.	16
Availability of data, code and other materials	27	Report which of the following are publicly available and where they can be found: template data collection forms; data extracted from included studies; data used for all analyses; analytic code; any other materials used in the review.	N/A

From: Page MJ, McKenzie JE, Bossuyt PM, Boutron I, Hoffmann TC, Mulrow CD, et al. The PRISMA 2020 statement: an updated guideline for reporting systematic reviews. BMJ 2021;372:n71. doi: 10.1136/bmj.n71

For more information, visit: http://www.prisma-statement.org/

S2

Scopus

"MTI*" OR "Magnetization Transfer Imaging*" OR " MT MRI*" AND "Spinal cord*" OR "cervical spinal cord*" OR "CSC*" AND "repeatability*" OR "reliability*" AND "reproducibility*"

Pubmed

" MTI *" OR " Magnetization Transfer Imaging *" OR " MT MRI MRI*" AND " cervical spinal cord *" OR "CSC*" AND "repeatability*" OR "reliability*" AND "reproducibility*"

Web of Science

"MTI*" OR " Magnetization Transfer Imaging *" OR " MT MRI*" AND " cervical spinal cord *" OR "CSC*" AND "repeatability*" OR "reliability*" AND "reproducibility*"

S3

QuADS Criteria	0	1	2	3
1. Theoretical or conceptual underpinning to the research	No mention at all.	General reference to broad theories or concepts that frame the study. e.g. key concepts were identified in the introduction section.	Identification of specific theories or concepts that frame the study and how these informed the work undertaken. e.g., key concepts were identified in the introduction section and applied to the study.	Explicit discussion of the theories or concepts that inform the study, with application of the theory or concept evident through the design, materials and outcomes explored. e.g., key concepts were identified in the introduction section and the application apparent in each element of the study design.
2. Statement of research aim/s	No mention at all.	Reference to what the sought to achieve embedded within the report but no explicit aims statement.	Aims statement made but may only appear in the abstract or be lacking detail.	Explicit and detailed statement of aim/s in the main body of report.
3. Clear description of research setting and target population	No mention at all.	General description of research area but not of the specific research environment e.g. 'in primary care.'	Description of research setting is made but is lacking detail e.g. 'in primary care practices in region [x]'.	Specific description of the research setting and target population of study e.g., 'nurses and doctors from GP practices in [x] part of [x] city in [x] country.'

4. The study design is appropriate to address the stated research aim/s	No research aim/s stated, or the design is entirely unsuitable e.g. a Y/N item survey for a study seeking to undertake exploratory work of lived experiences.	The study design can only address some aspects of the stated research aim/s e.g., use of focus groups to capture data regarding the frequency and experience of a disease.	The study design can address the stated research aim/s but there is a more suitable alternative that could have been used or used in addition e.g., addition of a qualitative or quantitative component could strengthen the design.	The study design selected appears to be the most suitable approach to attempt to answer the stated research aim/s.
5. Appropriate sampling to address the research aim/s	No mention of the sampling approach.	Evidence of consideration of the sample required e.g. the sample characteristics are described and appear appropriate to address the research aim/s.	Evidence of consideration of sample required to address the aim. e.g. the sample characteristics are described with reference to the aim/s.	Detailed evidence of consideration of the sample required to address the research aim/s. e.g. sample size calculation or discussion of an iterative sampling process with reference to the research aims or the case selected for study.
6. Rationale for choice of data collection tool/s	No mention of rationale for data collection tool used.	Very limited explanation for choice of data collection tool/s. e.g. based on availability of tool.	Basic explanation of rationale for choice of data collection tool/s. e.g. based on use in a prior similar study.	Detailed explanation of rationale for choice of data collection tool/s. e.g., relevance to the study aim/s, codesigned with the target population or assessments of tool quality.
7. The format and content of data collection tool is	No research aim/s stated and/or data collection tool not detailed.	Structure and/or content of tool/s suitable to address some aspects of the research aim/s or to address the aim/s	Structure and/or content of tool/s allow for data to be gathered broadly addressing	Structure and content of tool/s allow for detailed data to be gathered around all relevant issues required to

appropriate to address the stated research aim/s		superficially e.g., single item response that is very general or an open-response item to capture content which requires probing.	the stated aim/s but could benefit from refinement. e.g., the framing of survey or interview questions are too broad or focused to one element of the research aim/s.	address the stated research aim/s.
8. Description of data collection procedure	No mention of the data collection procedure.	Basic and brief outline of data collection procedure e.g. ‘using a questionnaire distributed to staff’.	States each stage of data collection procedure but with limited detail or states some stages in detail but omits others e.g. the recruitment process is mentioned but lacks important details.	Detailed description of each stage of the data collection procedure, including when, where and how data was gathered such that the procedure could be replicated.
9. Recruitment data provided	No mention of recruitment data.	Minimal and basic recruitment data e.g. number of people invited who agreed to take part.	Some recruitment data but not a complete account e.g. number of people who were invited and agreed.	Complete data allowing for full picture of recruitment outcomes e.g. number of people approached, recruited, and who completed with attrition data explained where relevant.

10. Justification for analytic method selected	No mention of the rationale for the analytic method chosen.	Very limited justification for choice of analytic method selected. e.g., previous use by the research team.	Basic justification for choice of analytic method selected e.g., method used in prior similar research.	Detailed justification for choice of analytic method selected e.g., relevance to the study aim/s or comment around of the strengths of the method selected.
11. The method of analysis was appropriate to answer the research aim/s	No mention at all.	Method of analysis can only address the research aim/s basically or broadly.	Method of analysis can address the research aim/s but there is a more suitable alternative that could have been used or used in addition to offer a stronger analysis.	Method of analysis selected is the most suitable approach to attempt answer the research aim/s in detail e.g., for qualitative interpretative phenomenological analysis might be considered preferable for experiences vs. content analysis to elicit frequency of occurrence of events.
12. Evidence that the research stakeholders have been considered in research design or conduct.	No mention at all.	Consideration of some the research stakeholders e.g., use of pilot study with target sample but no stakeholder involvement in planning stages of study design.	Evidence of stakeholder input informing the research. e.g., use of pilot study with feedback influencing the study design/conduct or reference to a project reference group established to guide the research.	Substantial consultation with stakeholders identifiable in planning of study design and in preliminary work e.g., consultation in the conceptualisation of the research, a project advisory group or evidence of stakeholder input informing the work.

13. Strengths and limitations critically discussed	No mention at all.	Very limited mention of strengths and limitations with omissions of many key issues. e.g. one or two strengths/limitations mentioned with limited detail.	Discussion of some of the key strengths and weaknesses of the study but not complete. e.g. several strengths/limitations explored but with notable omissions or lack of depth of explanation.	Thorough discussion of strengths and limitations of all aspects of study including design, methods, data collection tools, sample & analytic approach.
